# Predictors of COVID-19 severity among pregnant patients

**DOI:** 10.17305/bjbms.2022.7181

**Published:** 2022-06-05

**Authors:** Marcin Januszewski, Laura Ziuzia-Januszewska, Alicja A. Jakimiuk, Tomasz Oleksik, Marek Pokulniewicz, Waldemar Wierzba, Krzysztof Kozłowski, Artur J. Jakimiuk

**Affiliations:** 1 Department of Obstetrics and Gynecology, Central Clinical Hospital of the Ministry of the Interior and Administration, Warsaw, Poland; 2 Department of Otolaryngology, Central Clinical Hospital of the Ministry of Interior and Administration, Warsaw, Poland; 3 Department of Plastic Surgery, Central Clinical Hospital of the Ministry of the Interior and Administration, Warsaw, Poland; 4 University of Humanities and Economics in Lodz, Satellite Campus in Warsaw, Warsaw, Poland; 5 Department of Constitutional Law, Jagiellonian University in Krakow, Krakow, Poland; 6 Center for Reproductive Health, Institute of Mother and Child, Warsaw, Poland

**Keywords:** COVID-19, pregnancy, SARS-CoV-2, clinical course, predictors, disease severity, lymphocytopenia, hypocalcemia, low total protein, inflammation biomarkers

## Abstract

Coronavirus disease 2019 (COVID-19) was declared a pandemic and has spread around the globe, unsparingly affecting vulnerable populations. Effective prevention measures for pregnant women, who are particularly affected, include early identification of those patients at risk of developing in-hospital complications, and the continuous improvement of maternal-fetal treatment strategies to ensure the efficient use of health resources. The objective of our retrospective study was to determine which patient biomarkers on hospital admission correlate with disease severity as measured by disease course classification, the need for oxygen supplementation and higher demand for oxygen, the need for mechanical ventilation, intensive care unit admission, and length of hospital stay. Analysis of 52 PCR SARS-CoV-2 positive pregnant women revealed that the median date of hospital admission was the 30^th^ gestational week, with dyspnea, cough, and fever as the leading symptoms. The presence of diabetes and hypertension predisposed pregnant women to the severe course of illness. Lung involvement shown by CT scans on admission correlated with the greater clinical severity. The main laboratory predictors of disease progression were lymphocytopenia, hypocalcemia, low total cholesterol, low total protein levels, and high serum levels of C-reactive protein, ferritin, interleukin-6, glucose, lactate dehydrogenase, procalcitonin, and troponin I. Further, research with a larger cohort of pregnant women is needed to determine the utility of these results for everyday practice.

## INTRODUCTION

Coronavirus disease 2019 (COVID-19) caused by severe acute respiratory syndrome coronavirus 2 (SARS-CoV-2) is widespread and globally claims more victims with each passing month [1]. As more alarming data about the characteristics of new SARS-CoV-2 variants emerge and as we witness the natural evolution and increased infection rates of COVID-19, it is increasingly important to be able to prioritize critical care services in situations, where the number of patients may be overwhelming. Prenatal care, which is particularly affected, deserves special attention and continuous improvement of its treatment strategies.

The distribution of disease severity in pregnant women is similar to the distribution seen in non-pregnant populations, with 86% of pregnant women manifesting mild disease, 9% severe, and 5% critical [2].

SARS-CoV-2 affects nearly every organ system [3-7], as well as affecting the mental health of both infected and non-infected pregnant women [8]. Leading symptoms include fever (88.7%), cough (67.8%), fatigue (38%), and the over-production of mucus (33.7%) [4]. Severe COVID-19 is characterized by the development of acute respiratory distress syndrome (ARDS), hypotensive shock, and multiorgan failure and requires the patient’s admission to intensive care unit (ICU) and mechanical ventilation [3-5].

Severe disease risk factors include comorbidities, advanced age, male sex, obesity, and genetic predispositions [3,4,5,9]. Severe COVID-19 is mainly an immune-mediated disorder triggered by the SARS-CoV-2 infection promoting excessive inflammation and hypercoagulable states [10].

During pregnancy, physiological adaptations of the respiratory tract, immunomodulation, hypercoagulability, processes that increase insulin resistance, and the development of hypertension, predispose SARS-CoV-2-infected women toward a severe course of illness, leading to maternal and fetal mortality and morbidity [10-14].

Data emerging from meta-analyses in the literature show that pregnant women may have an increased risk of developing severe symptoms and a higher risk of pneumonia, ICU admission, the requirement for invasive ventilation and extracorporeal membrane oxygenation (ECMO), and death [15-17].

Moreover, serious adverse outcomes have been observed among pregnant women with previous coronavirus infections, namely, severe acute respiratory syndrome (SARS) and Middle East respiratory syndrome (MERS) [18], influenza [19,20], and respiratory syncytial virus [21].

There is currently no prognostic biomarker available to identify pregnant patients who are at imminent risk of a severe course of COVID-19, with all associated maternal and fetal complications, and who require immediate medical attention.

The objective of our study was to determine to determine, in which patient characteristics and laboratory results on hospital admission correlate with disease severity as measured by disease course classification, the need for oxygen supplementation and higher demand, the need for mechanical ventilation, ICU admission, and length of hospital stay.

## MATERIALS AND METHODS

### Study population

This retrospective single-center study was undertaken in the Department of Obstetrics and Gynecology, at the Central Clinical Hospital of the Ministry of the Interior and Administration in Warsaw, Poland. The study group comprised 52 pregnant women with COVID-19 who had been admitted for treatment between 15 May 2020 and 26 April 2021.

Inclusion criteria, similar to admission indications for pregnant women with COVID-19, were temperature >39°C despite the use of acetaminophen, respiratory rate >30/min, SpO_2_ <95% measured at time of admission without oxygen supplementation, patient requiring oxygen, and critical disease. COVID-19 was confirmed using a PCR test prior to admission.

Exclusion criteria were that the patient was admitted to hospital for obstetric and/or other non-COVID-19-related reasons.

### Clinical course of the disease

According to the guidelines of the Polish Association of Epidemiologists and Infectiologists, patients were divided into four cohorts based on the severity of their symptoms and test results, corresponding to the relative course of their illness: mild, moderate, severe, and critical [22].

Mild cases were characteristically clinically stable with mild upper respiratory tract symptoms. Moderate cases included clinical indicators as well as lung involvement shown on imaging. Patients in the severe cohort demonstrated respiratory failure and peripheral SpO_2_ <90%. Those in the critical cohort were characterized by ARDS, hypotensive shock, multiorgan failure, and loss of consciousness [22].

### Study procedures

On admission, all women underwent complete blood biochemistry and urine tests, a coagulation profile, and in cases where moderate, severe, or critical forms of COVID-19 were suspected, a CT chest scan (without contrast) was performed.

We analyzed the following data: age of patient, body mass index (BMI), gestational age, initial vital signs and symptoms, pre-existing comorbidities such as diabetes mellitus, hypertension, hypothyroidism, asthma, and any history of smoking.

### Ethical statement

The research project was approved by the local Bioethics Committee (Decision Number104/2021).

### Statistical analysis

We used Statistica 13.3 (StatSoft Poland) for our data analysis. Mean values and standard deviations were used to describe the study groups. In case of skewed distributions, the median was calculated as a measure of central tendency, and the scatter of data was shown in relation to the 25^th^ and 75^th^ percentiles. Qualitative variables were presented as percentages. Spearman’s rank correlation was used to assess correlation. In case of qualitative variables, the Chi-square test was used to compare the frequencies of the studied characteristics. Differences were considered statistically significant at *p* < 0.05. Logistic regression was performed to analyze the association of patient characteristics and laboratory parameters and the risk of severe-to-critical disease. Non-linear data were categorized. Variables with more than 20% of their values missing were not considered in this analysis, in other cases, missing values were analyzed as a separate category. Comparisons between the four disease severity groups were performed using the Kruskal–Wallis test followed by pair-wise comparison using Dunn’s *post hoc* test for continuous variables, and Pearson’s chi-square test for categorical variables. As there was only one patient with a critical course of the disease, the severe and critical groups were combined in the analysis as a new grouping, severe-to-critical disease.

## RESULTS

Gestational age ranged from 17 to 37 weeks. Four patients were at 17-22 weeks of gestation, 14 were at 24-28, 17 were at 29-33, 15 were at 34-36, and 2 patients were at >37 weeks of gestation. The mean age of the patients was 31.9 ± 4.79 years. Mean BMI at admission was 28.36 (9.88) kg/m^2^. None of the patients reported a history of smoking. Symptoms on admission were: dyspnea (n = 48, 92.31%), cough (n = 47, 90.38%), fever (n = 33, 63.46%), fatigue and muscle aches (n = 22, 42.31%), smell and taste disorders (n = 14, 26.92%), headache (n = 12, 23.08%), sore throat (n = 6, 11.54%), and nasal discharge (n = 5, 9.62%). Coexisting diseases were diabetes (n = 9, 17.65%), hypertension (n = 5, 10.00%), hypothyroidism (n = 18, 35.29%), and asthma (n = 2, 3.85%). Mild, moderate, severe, and critical COVID-19 accounted for n = 9 (17.31%), n = 25 (48.08%), n = 17 (32.69%), and n = 1 (1.92%) cases, respectively. The main outcomes measured were length of hospitalization (median = 8 [range = 2-23] days), the need for oxygen supplementation (n = 42, 80.77%), median oxygen flow rate (median = 4 [range = 0-15]), requirement for high-flow therapy (n = 9, 17.31%), and the need for ICU admission (n = 2, 3.85%). There were no cases of tracheal intubation, mechanical ventilation, or ECMO. The median lung involvement seen by CT imaging was 20% (IQR = 11), ranging from 1% to 60%. The most common abnormalities shown in the laboratory results that were elevated C-reactive protein (CRP) (94.23%), elevated D-dimer (90.63%), elevated interleukin 6 (IL-6) (88.46%), elevated fibrinogen (88%), hypoproteinemia (66.67%), decreased vitamin D (62.22%), elevated lactate dehydrogenase (LDH) (56%), hyperglycemia (48.78%), anemia (48.08%), elevated alkaline phosphatase (ALP) (46.15%), elevated aspartate aminotransferase (AST) (40.38%), lymphopenia (38.46%), neutrophilia (30.77%), elevated alanine transaminase (ALT) (30%), and elevated bile acids (35.71%). Data regarding patients’ characteristics, clinical course parameters, and laboratory abnormalities are presented in Table 1.

**TABLE 1:**
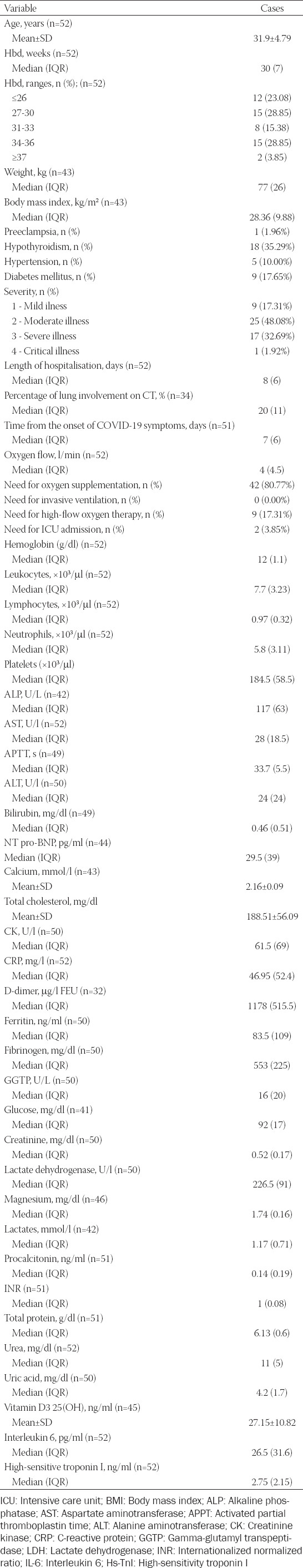
Clinical characteristics of 52 pregnant COVID-19 patients

### Main predictors of severe course of illness

Diabetes as a comorbidity was correlated with the need for high-flow oxygen therapy and higher oxygen flow. Hypertension was correlated with oxygen flow demand during hospitalization. The percentage of lung involvement correlated with four of the six main outcomes: the severity of the course of the COVID-19, the oxygen flow (l/min), the need for high-flow oxygen therapy, and the need for ICU admission (Table 2).

**TABLE 2:**
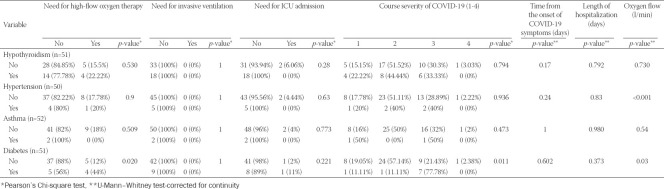
Correlation of comorbidities and COVID-19 severity

Lymphocytopenia, low levels of serum calcium, total cholesterol and total protein levels, high levels of serum CRP, ferritin, IL-6 glucose, LDH, procalcitonin (PCT), and high-sensitivity (hs) troponin I predicted a severe course of illness as measured by disease course classification, the need for oxygen supplementation, higher demand for oxygen supplementation, length of hospital stay, the need for mechanical ventilation, and ICU admission. The results are presented in Table 3. Patients’ characteristics and laboratory markers compared across four severity categories are presented in Table 4. Univariate logistic regression revealed that diabetes (odds ratio [OR] 10.18, 95% CI 1.83-56.54; *p* = 0.008), gestational age < 32 weeks (OR 5, 95% CI 1.36-18.43; *p* = 0.016), lung involvement on CT imaging > 20% (OR 5.8, 95% CI 1.54-21.81, *p* = 0.009), lymphocyte count < 1(x10^3^/μl) (OR 27.43, 95% CI 3.26-231.58; *p* = 0.002), calcium level ≤ 2.15 (mmol/l) (OR 5.56, 95% CI 1.61-19.22; *p* = 0.007), CRP > 75(mg/l) (OR 9.11, 95% CI 2.38-34.85; *p* = 0.001), IL-6 > 60 (pg/ml) (OR 16.5, 95% CI 1.8-151.58; *p* = 0.013), procalcitonin > 0.2(ng/ml) (OR 5.11, 95% CI 1.49-17.56; *p* = 0.010), LDH > 270 (U/l) (OR 3.73, 95% CI 1.04-13.45; *p* = 0.044), total cholesterol ≤ 180 (mg/dl) (OR 5.65, 95% CI 1.53-20.93; *p* = 0.010), total protein level ≤ 6.3 (g/dl) (OR 9, 95% CI 1.79-45.34; *p* = 0.008), hs-troponin I > 6 (ng/ml) (OR 12.69, 95% CI 1.35-119.34), and glucose > 99 (mg/dl) (OR 6, 95% CI 1.48-24.27; *p* = 0.012) were associated with increased risk of severe-to-critical COVID-19.

**TABLE 3:**
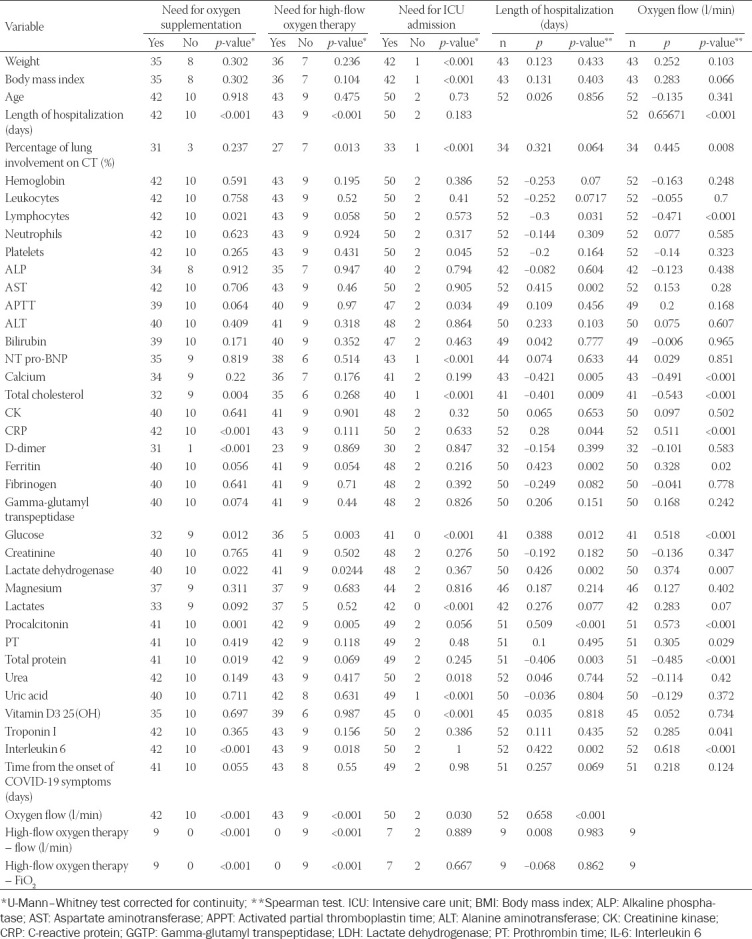
Correlations of patient`s general and clinical characteristics and COVID-19 outcomes

**TABLE 4:**
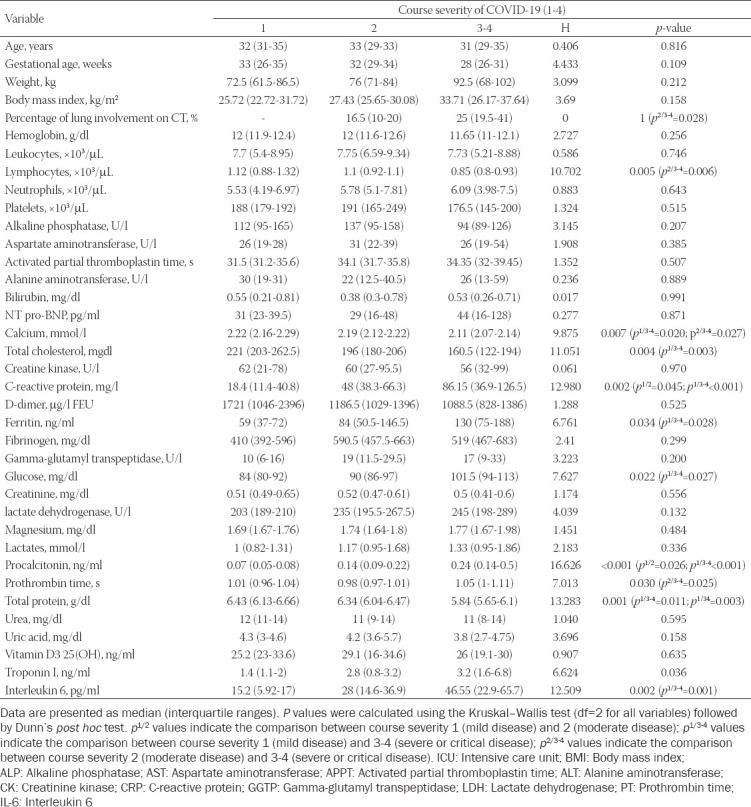
Association of patients’ characteristics and COVID-19 severity

## DISCUSSION

In our study, the median date of the pregnant women’s hospital admission was the 30^th^ gestational week (range = 17-37^th^ week). Because COVID-19 is an immune-mediated intracellular viral infection, it may pose a threat during pregnancy due to the special immunological adaptations that improve a pregnant woman’s tolerance to the fetal semi-allograft late in the second trimester and the increased inflammatory response in the third trimester [10,14,23-25]. In addition, hypertension, diabetes, and cardiovascular diseases that develop during the third trimester may predispose pregnant women to the severe course of illness. Therefore, we advise vaccination in the second trimester for maternal and fetal benefits [26].

We found that median lung involvement was 20%, with a range of 1-60%, and as lung involvement at the time of admission correlated with 4 of the 6 main outcomes – the severity of the course of the COVID-19 disease, oxygen flow (l/min), the need for high-flow oxygen therapy, and the need for ICU admission – lung involvement may be considered as a predictor of disease aggravation. Based on the pathophysiology of COVID-19 progression, it seems like a truism to claim that the greater lung involvement is, the greater the severity of the disease, which is corroborated in our findings. However, the previous studies in pregnant women report contrasting results: One indicating greater lung involvement among pregnant women, and another reporting that lung involvement was similar in both pregnant and non-pregnant subjects [27,28].

Several previous studies have attempted to determine which laboratory parameters correlate with disease severity among pregnant women with COVID-19. Those studies found that subjects’ laboratory results largely mirrored those in the adult non-pregnant population, especially regarding lymphopenia and inflammation parameters.

Severe COVID-19 is associated with higher levels of inflammatory markers than in mild disease. Therefore, tracking these markers may permit early identification of patients at risk of disease progression. Likewise, a link between increased cardiac markers and disease aggravation with a few potential pathomechanisms is well established in the literature [29]. COVID-19 can cause direct or indirect heart injury: cardiomyocyte viral infection, cytokine-mediated systemic inflammation, supply-demand mismatch, and micro- and macrovascular thrombosis [29-31].

Lymphopenia has been identified as the most distinctive predictive parameter [32-34]. In a study by Lombardi et al., lymphocyte values at admission correlated with the oxygen need. CRP levels were found to be the inflammatory biomarker that better mirrored the course of the disease than D-dimer or ferritin levels, which were not reliable predictors of a poor outcome [32]. The retrospective study of 217 pregnant women with COVID-19 by Bozkurt et al. showed that elevated LDH, CRP, IL-6, and ferritin levels coupled with low albumin levels on hospital admission were predictive parameters for a more severe course of illness, and that elevated serum levels of blood urea nitrogen and creatine were the most predictive parameters for ICU admission [35]. Data support that the host’s immune system overreaction (cytokine storm syndrome or cytokine release syndrome) may play an important role in the pathogenesis of SARS [36]. SARS-CoV infection may lead to hyper-induction of the immune system, causing increased levels of cytokines, e.g., IL-6 and chemokines, all of which have been observed in SARS patients. However, there are also contradicting results [37]. Data have shown that IL-6 levels are also significantly higher in COVID-19 patients with severe disease compared with those with a non-severe condition. Therefore, IL-6 is a prognostic marker for serious COVID-19 cases in pregnant [38] and non-pregnant cohorts [39-41].

Our study identified a positive correlation between exact glucose values at admission and poorer patient outcomes. This observation suggests that the elevated blood sugar levels we observed may be the result of physiological stress triggered by the disease. COVID-19 disrupts glucose regulation, rendering poor glycemic control, and necessitating particularly careful management in patients with diabetes [42,43]. Indeed, prior work has shown that even in cases of well-controlled pre-existing diabetes, hyperglycemia was commonly observed in acutely ill hospitalized patients and linked to adverse outcomes [44,45]. It seems that COVID-19 may lead to high blood glucose levels in patients with normal glycemic status by modulating immune and inflammatory responses, directly affecting morbidity and mortality [46-48]. In a study by Charoenngam et al. in patients without a history of diabetes, hyperglycemia on the day of admission was shown to have a statistically significant association with mortality, ICU admission, intubation, acute kidney injury, and severe sepsis/septic shock, after adjusting for potential confounders. Therefore, it could be a strong indicator of a high inflammatory burden, leading to a higher risk of severe COVID-19 [49]. Thereby, we recommend that clinicians pay more attention to the blood glucose status of pregnant women with COVID-19, even those who may not have been diagnosed with diabetes prior to admission.

In our study, calcium serum levels were negatively correlated with three measured clinical outcomes: the length of hospitalization (days), the severity of the course of COVID-19 (1-4), and oxygen flow (l/min). These findings were consistent with previously published reports which have showed that low serum calcium levels are associated with disease severity and a poor prognosis for patients with COVID-19 [50-53]. In a study by Zhang et al. low serum calcium levels were the most predictive feature of COVID-19 diagnosis of all models tested [54]. The cause of hypocalcemia in COVID-19 patients is not clear. It is commonly found in the laboratory results in patients diagnosed with viral infections and pneumonia [55], and several mechanisms may be suggested. Firstly, the pro-inflammatory cytokines in COVID-19 patients inhibit parathyroid hormone (PTH) secretion, and the resulting impaired response to PTH causes an imbalance of calcium levels [56]. According to the previous studies, levels of the disease progression indicators CRP, PCT, IL-6, and D-dimer are found to be significantly higher in COVID-19 patients with hypocalcemia. When this is coupled with calcium serum levels which are negatively correlated with these indicators, it means that these patients may have a greater inflammatory response [50,51,53]. Secondly, the occurrence of hypocalcemia may be associated with calcium inflow due to hypoxemic tissue damage. Another theory is that modification of calcium levels is crucial for the survival and replication of the SARS-CoV-2, since calcium is used in virus structure formation, entry, gene expression, virion maturation, and release [57]. Pregnancy also often leads to vitamin D deficiency resulting in hypocalcemia due to impaired intestinal absorption and thus an inadequate intake of calcium [58]. Finally, calcium is predominantly bound to albumin in plasma, and a decrease in serum albumin or total protein levels, mainly occurring in the third trimester, will cause hypocalcemia [59].

Our work showed that low total protein serum levels are a predictive factor for both a longer time to clinical improvement and a greater severity of disease, and as such this predictor can provide useful information for clinicians caring for pregnant women with COVID-19. Several mechanisms were proposed, including anti-oxidative and anti-inflammatory values of albumin [60,61], downregulation of albumin and prealbumin caused by the cytokine storm [62], and dysregulation of the immune system triggered by low protein serum levels [63]. The previous studies have indicated that serum albumin [64-66], prealbumin [62], and total protein levels are poor prognosis parameters among non-pregnant cohorts [63]. As mentioned earlier, total protein and albumin levels decrease because of the physiological processes of pregnancy during the third trimester. This condition poses a major threat to pregnant SARS-CoV-2 infected women as outlined in a study by Bozkurt et al. [35].

Our study showed that on admission, disrupted total cholesterol levels correlate with a greater severity of disease and with five out of the six main outcomes: the length of hospitalization (days), the severity of the course of COVID-19 (1-4), oxygen flow (l/min), the need for oxygen supplementation, and the need for ICU admission. The role of cholesterol in immunity is well established in numerous observational studies. In addition, dynamic changes in lipid levels caused by SARS-CoV-2 might be explained by several hypotheses. Firstly, the production of apolipoproteins and lipoproteins might be impaired by liver damage [67] and cytokine activity [68], and secondly, capillary leakage may occur, relocating them to extravascular compartments [69]. A study by Wei et al. demonstrated that patients with COVID-19 develop hypolipidemia during early stages of the disease and that abnormalities in lipid metabolism progressively became worse in association with the severity of the disease [70]. Lower levels of total cholesterol, low-density lipoprotein, and high-density lipoprotein (HDL) were linked to higher mortality rates and poorer prognoses in patients with COVID-19 [70-73]. Moreover, high CRP/HDL-C ratios were established as an independent predictor of in-hospital mortality [74]. However, none of these previously published studies involved pregnant women.

The main limitations of our investigation include its single-center nature, as well as its small and homogeneous cohort of patients. Moreover, we evaluated markers on admission, and not their response to disease progression and treatment.

## CONCLUSION

Pregnant women with COVID-19 were hospitalized during their second or third trimester, with dyspnea, cough, and fever as leading symptoms. Concomitant conditions, including diabetes and hypertension, modified the course of illness. CT chest scan at initial presentation may enable medical services required by pregnant patients infected with SARS-CoV-2 to be prioritized. Lymphocytopenia, hypocalcemia, low total cholesterol, low total protein levels, and high serum levels of CRP, ferritin, IL-6, procalcitonin, hs-troponin I, LDH, and glucose measured on hospital admission are good predictors of disease severity and may lead to early identification of patients at risk for developing complications, thereby improving optimization and prevention efforts in this cohort. Further, research with a larger patient sample and risk models is needed to provide useful information for effective health resource management during the COVID-19 pandemic.
